# Linking Cancer Pain Features and Biosignals for Automatic Pain Assessment

**DOI:** 10.3390/cancers18040646

**Published:** 2026-02-16

**Authors:** Marco Cascella, Francesco Perri, Alessandro Ottaiano, Mariachiara Santorsola, Maria Luisa Marciano, Fabiana Raffaella Rampetta, Monica Pontone, Anna Crispo, Francesco Sabbatino, Gianluigi Franci, Walter Esposito, Gennaro Cisale, Maria Romano, Francesco Amato, Amalia Scuotto, Vittorio Santoriello, Alfonso Maria Ponsiglione

**Affiliations:** 1 Department of Medicine, Surgery and Dentistry, University of Salerno, 84081 Baronissi, Italy; mcascella@unisa.it (M.C.);; 2 Head and Neck Oncology Unit, Istituto Nazionale Tumori di Napoli IRCCS “G. Pascale”, 80131 Naples, Italy; ml.marciano@istitutotumori.na.it (M.L.M.); fabianaraffaella.rampetta@istitutotumori.na.it (F.R.R.);; 3 Sarcomas and Rare Tumors Unit, Istituto Nazionale Tumori, IRCCS—Fondazione “G. Pascale”, 80131 Naples, Italy; a.ottaiano@istitutotumori.na.it; 4 Istituto Nazionale Tumori-IRCCS “Fondazione G. Pascale”, 80131 Naples, Italy; 5 Department of Electrical Engineering and Information Technology, University of Naples Federico II, 80125 Naples, Italy

**Keywords:** cancer pain, breakthrough cancer pain, biosignals, electrodermal activity, automatic pain assessment, heart rate variability

## Abstract

**Background:** Pain remains one of the most debilitating and prevalent symptoms in cancer patients. However, assessment based solely on subjective self-report tools is limited by cognitive impairment and the heterogeneous nature of cancer pain. Since evidence on the ability of physiological biosignals to discriminate cancer pain intensity and pain phenotypes in real clinical settings remains limited, this study explored the potential of biosignals to discriminate between pain intensity and pain type. **Methods:** Electrodermal activity (EDA) and electrocardiogram (ECG) signals were recorded in cancer patients using the BITalino (r)evolution board (sampling frequency 1000 Hz). EDA was processed to extract skin conductance responses (SCRs) using continuous decomposition analysis (CDA) and trough-to-peak (TTP) methods. Heart rate variability (HRV) features were extracted in both time and frequency domains, including low frequency (LF), high frequency (HF), and the LF/HF ratio. Non-parametric Kruskal–Wallis tests were performed to compare biosignal parameters across pain intensity (Numeric Rating Scale, NRS: low 1–3; medium 4–6; and high 7–10) and pain types (nociceptive, neuropathic, mixed, and breakthrough cancer pain—BTCP). **Results:** Data from 61 patients were analyzed. For EDA, the maximum skin conductance response amplitude (MaxCDA) significantly differed across intensity groups (*p* = 0.037). Post hoc analysis showed a significant difference between the low- and high-intensity groups (*p* = 0.015), with the low-intensity group exhibiting a higher mean MaxCDA (0.063 µS) than the high-intensity group (0.024 µS). Several EDA parameters were significantly associated with pain type. The number of SCRs (TTP) (*p* = 0.015) and maximum SCR amplitude (TTP) (*p* = 0.040) were significantly lower in the mixed pain group compared with the nociceptive and neuropathic groups. No HRV parameters showed significant associations with pain intensity or pain type. BTCP did not significantly affect any biosignal parameters. Subgroup analyses showed that EDA features discriminating mixed pain were preserved in patients without bone metastases, BTCP, or high opioid burden, whereas no clinical variable modified the association between biosignals and pain intensity and type. **Conclusions:** In this investigation, selected EDA parameters were associated with cancer pain intensity and pain type, whereas heart rate variability measures did not show significant discrimination under the present methodological conditions. These findings suggest that EDA may provide complementary information on pain-related autonomic alterations in oncology patients. However, biosignals should not be considered standalone indicators of pain, and their interpretation requires integration with clinical variables and pharmacological context. Further studies adopting multimodal and longitudinal approaches are needed to clarify their role in automatic pain assessment in cancer care.

## Simple Summary

Although pain is a frequent and burdensome symptom in people with cancer, it is commonly evaluated using self-reported scales that may be unreliable in patients with cognitive, communicative, or clinical limitations. This study explored whether objective physiological signals could enhance cancer pain assessment. We analyzed electrodermal activity and heart rate variability recorded in cancer patients and examined their relationships with pain intensity and pain type. The results indicate that specific electrodermal activity parameters are associated with both pain intensity and distinct pain phenotypes (mainly mixed pain). In contrast, heart rate variability failed to provide meaningful discrimination in this context. Despite limitations, these findings suggest that electrodermal activity may represent a valuable objective marker to complement conventional pain scales and support the development of automated pain assessment approaches in oncology.

## 1. Introduction

Pain is one of the most frequent and distressing symptoms experienced by individuals with cancer. Despite well-established clinical guidelines, significant gaps persist in the management of pain in this vulnerable population [1,2]. Consequently, many patients, particularly those in advanced or terminal disease stages, continue to experience moderate to severe pain. Systematic reviews have reported that pain prevalence in patients with advanced or metastatic cancer may reach 64–75%, often with high intensity levels [3,4].

Obtaining an accurate pain diagnosis remains a key barrier to effective management [2]. Cancer pain encompasses a broad and heterogeneous range of painful conditions with diverse physiological and clinical characteristics, including nociceptive, neuropathic, and mixed pain, as well as breakthrough cancer pain (BTCP) [5]. Furthermore, reliance on subjective, self-report tools such as the Numeric Rating Scale (NRS) is inherently limited by factors such as cognitive impairment, communication difficulties, cultural variability, and the inherently subjective nature of pain [6].

These limitations have driven growing interest in objective, physiology-based methods to complement traditional assessment tools. In automatic pain assessment (APA), behavioral strategies and physiological signals—also known as biosignals—are used to evaluate and monitor pain [7]. Specifically, biosignals, including electrodermal activity (EDA), electroencephalography (EEG), and electrocardiography (ECG)-derived heart rate variability (HRV), provide quantifiable, non-verbal indicators of autonomic nervous system (ANS) activity that correlate with pain-related arousal [8,9,10]. Importantly, published studies have explored the use of these biosignals as objective markers of pain, mainly within experimental paradigms or controlled laboratory settings. For example, pressure stimuli applied to limbs elicited pain-dependent changes in EDA and HRV in healthy participants, demonstrating associations between autonomic responses and pain intensity levels [11]. Furthermore, a scoping review on wearable sensors also highlighted the potential of HRV and EDA to capture pain-related physiological responses in controlled conditions where self-report may be limited [12]. In a systematic review, Forte et al. [13] reported that heart rate variability may serve as a useful marker for assessing nociceptive responses in experimentally induced pain. However, the authors also emphasized the need for further studies in clinical populations to better characterize interindividual variability in autonomic responses to pain stimuli.

In oncology, emerging evidence suggests that ANS dysregulation plays a relevant role in chronic cancer pain; however, results remain heterogeneous and strongly influenced by clinical context, disease burden, and pharmacological treatments [14]. Systematic and narrative reviews underscore the significant gap in real-world clinical evidence, highlighting that most APA studies in cancer patients lack external validity, are often limited to single-modality approaches, and fail to adequately capture the complexity of chronic pain phenotypes [7,15]. In other words, recognizing that pain is a multifaceted and subjective experience, the automated measurement of physiological responses to nociception detects only part of this complex phenomenon [10,16]. Nevertheless, objective signal-based assessment methods may help overcome key limitations of traditional pain evaluation strategies [17].

In a previous study, we investigated biosignals alongside demographic, clinical, and pain-related variables to develop a multidimensional analytical framework [18]. The present work builds on that foundation to perform an in-depth analysis of biosignals. Specifically, it further explores autonomic nervous system (ANS) responses to pain in cancer patients using multiple signal processing techniques to identify physiological markers associated with pain intensity and type. Therefore, this study aimed to investigate whether objective physiological signals, specifically EDA and HRV, are associated with cancer pain intensity and pain type, and to explore their potential role as biomarkers for APA in oncology. To address this issue, we employed a wearable biosignal acquisition system (BITalino (r)evolution board), a platform previously used for high-resolution electrodermal and cardiac signal acquisition in cancer pain research [8].

## 2. Methods

### 2.1. Study Design and Population

This study represents a post hoc analysis of data derived from a previously approved and registered investigation and was conducted in accordance with the ethical standards of the Declaration of Helsinki and its subsequent amendments, or comparable ethical standards. The study protocol received approval from the local Ethics Committee of the Istituto Nazionale Tumori, Fondazione Pascale, Naples, Italy (Protocol Code 41/20 Oss; approved on 26 November 2020). Furthermore, the study was prospectively registered on ClinicalTrials.gov (NCT04726228) and all participating adults provided written informed consent before inclusion.

### 2.2. Participants

Patients were consecutively recruited. Eligibility criteria included age ≥ 18 years, a confirmed diagnosis of solid cancer, the presence of chronic cancer-related pain lasting at least three months, the ability to communicate effectively and provide written informed consent, and completion of the biosignal acquisition. Exclusion criteria comprised severe cognitive impairment, psychiatric or neurological conditions known to affect ANS function, the presence of implanted cardiac devices, and clinically significant cardiac arrhythmias.

### 2.3. Data Collection

For each participant, a comprehensive set of clinical variables was collected, including demographic data, tumor type, baseline pain intensity (0–10 NRS) and type (nociceptive, neuropathic, mixed), BTCP (assessed at baseline as a dichotomous variable: yes/no), opioid therapy (yes/no), and Eastern Cooperative Oncology Group (ECOG) performance status. Furthermore, the presence of bone metastases and opioid intake (expressed as morphine equivalent dose, MED, less than or more than 60 mg/day) was also reported.

### 2.4. Biosignals

**Figure 1:**
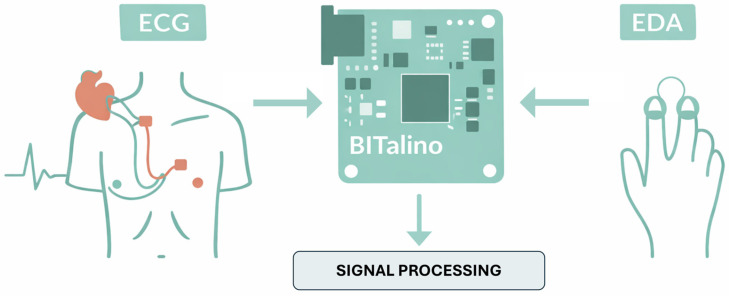
Biosignal acquisition and processing pipeline. Electrocardiogram (ECG) and electrodermal activity (EDA) signals are recorded using the BITalino (r)evolution board through dedicated sensors. The acquired raw physiological signals are then forwarded to the signal processing stage for feature extraction and subsequent analysis related to cancer pain phenomenology.

ECG and EDA signals were recorded using the BITalino (r)evolution Board (PLUX Wireless Biosignals, Lisbon, Portugal)., equipped with dedicated ECG and EDA sensors and capable of sampling up to 1000 Hz as reported in [8]. ECG was acquired using a single-lead Einthoven configuration with a triodal electrode setup, enabling subsequent extraction of inter-beat intervals and heart rate parameters. EDA was recorded from the fingers using biosignalsplux sensors (PLUX Wireless Biosignals, Lisbon, Portugal), designed to detect tonic variations and phasic skin conductance responses (SCRs) (Figure 1).

For processing, all signals were trimmed to the same length, corresponding to the shortest recording, to allow uniform processing. EDA signals were processed through continuous decomposition analysis (CDA) and trough-to-peak (TTP) analysis [19]. CDA performs a precise deconvolution of the raw SC data. This approach separates the total SC signal into two continuous, orthogonal components, including the tonic and phasic skin conductance activity (SCA). The tonic component represents the slow-moving baseline and reflects the general, sustained state of sympathetic activation (arousal level). The phasic component is the transient fluctuation, known as the skin conductance response (SCR), which reflects event-related or stimulus-driven sympathetic arousal. The CDA approach is considered superior for its ability to provide continuous and simultaneous estimates of both underlying components, offering a more robust quantification of sympathetic driver activity. The TTP method is a standard discrete measurement approach, often referred to as Min-Max analysis. It quantifies the amplitude of individual SCRs by calculating the difference between the peak SC value of a response and its preceding minimum (trough) value within pre-defined temporal windows specific to the stimulus onset. While the TTP method is conventional, it is recognized as being less precise than the CDA because it relies on the discrete identification of minima and maxima rather than a continuous deconvolution of the signal. For each SCR, the amplitude (maximum, minimum, and mean), the number of responses, and onset interval metrics (maximum, minimum, and mean) were computed, yielding fourteen EDA-related features for each patient.

The HRV analysis began with the ECG signal, employing an adapted version of the Pan–Tompkins algorithm to accurately detect the R-peaks. Subsequently, the RR interval series, representing the inter-beat interval (IBI), was calculated as the temporal difference between consecutive R-peaks. The RR series was then processed to derive characteristic HRV features across both time and frequency domains, as per standard guidelines [20]. Time-domain features, designed to quantify overall variability and short-term regulation, included the average heart rate, the Standard Deviation of NN intervals (SDNN), and the Root Mean Square of Successive Differences in the RR intervals (RMSSDs). For the frequency-domain assessment, Power Spectral Density (PSD) was estimated using Welch’s periodogram method. The resulting power was segmented across the following standard frequency bands: Low Frequency Power (LF) (0.04–015 Hz) and High Frequency Power (HF), spanning 0.15–0.36 Hz. Finally, the LF/HF ratio was calculated, providing a critical index of the sympatho-vagal balance [21]. Signal processing was performed in MATLAB v. R2022b.

As far as the statistical analyses, the following two sets of comparisons were carried out: (i) across pain intensity categories according to the NRS (mild: NRS 1–3; moderate: 4–6; and severe: 7–10), and (ii) across pain type (nociceptive, neuropathic, and mixed). Group comparisons were performed using the Kruskal–Wallis test, as a nonparametric alternative to the ANOVA test, due to the non-normal distribution of the biosignal features across the groups (either by baseline pain intensity or baseline pain type) as assessed through the Shapiro–Wilk normality test (features were found non-normally distributed in at least one subgroup). Post hoc pairwise comparisons were carried out using Dunn’s test. Given the exploratory nature of the study, both raw *p*-values (no adjustment) and Benjamini–Hochberg-adjusted *p*-values were considered in the post hoc pairwise comparisons. Finally, subgroup analyses were carried out using the Kruskal–Wallis test followed by Dunn’s post hoc test with Benjamini–Hochberg correction to examine the statistical significance of biosignal features across pain intensity and type while considering specific clinical variables (bone metastases, BTCP, and MED). All statistical analyses were conducted using IBM SPSS Statistics v.28 (IBM Corp, Armonk, New York, USA) and R software version 4.4.1 using RStudio (v. 2024.04.2, RStudio: Integrated Development for R. RStudio, Inc., Boston, MA, USA).

## 3. Results

A total of 128 patients were initially assessed for study eligibility. Of these, biosignal acquisition was successfully performed on 64 individuals. The median age was 61 years (SD = 13.3), with a male predominance (64.0%). The demographic and clinical profiles (considering cancer type, baseline pain intensity and type, BTCP, ECOG performance status, bone metastases, and MED of opioids) of the final cohort are summarized in Table 1.

The overall results are presented, detailing the statistical associations between biosignal features (EDA and HRV) and the clinical characteristics of cancer pain, specifically intensity, type, and the presence of BTCP.

### 3.1. Association with Pain Intensity

As far as the association between biosignals’ features and baseline pain intensity, as measured with the NRS scale, a statistically significant overall effect for pain intensity only for one EDA parameter, namely the maximum SCR amplitude derived from the CDA decomposition approach (“maxSCR_CDA”) (*p* = 0.037).

**Figure 2:**
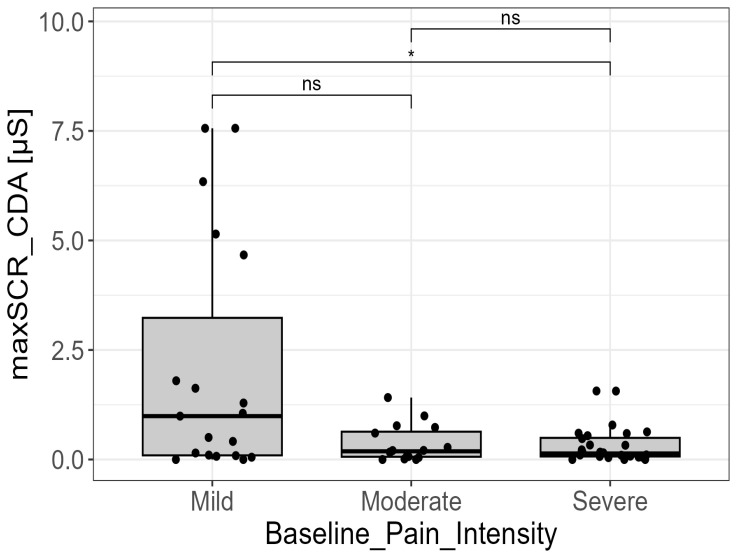
EDA signal and pain, as measured by NRS. Distribution of the maximum amplitude of skin conductance responses derived from continuous decomposition analysis (maxSCR_CDA) across baseline pain intensities. Statistical significance was assessed using Kruskal–Wallis test followed by post hoc pairwise comparisons (Dunn’s test). Only for visualization purposes, extreme outliers were removed from the graph. (ns = not significant; * = *p*-value < 0.05).

Concerning links between biosignal (i.e., the best parameter) and pain intensity, post hoc analysis (Dunn’s test) revealed that the significant difference in MaxCDA was specifically between the mild-intensity group and the severe-intensity group (*p* = 0.015; adjusted-*p* = 0.045). Patients in the mild-intensity group exhibited the highest median “maxSCR_CDA” (median = 1.02 µS; IQR = 4.69), which was substantially higher than the median observed in the severe-intensity group (median = 0.14 µS; IQR = 0.42). The moderate-intensity group (NRS 4–6) showed an intermediate response (median = 0.19 µS; IQR = 0.57) (Figure 2).

Maximum skin conductance response amplitude derived from continuous decomposition analysis (“maxSCR_CDA”) across pain intensity categories according to the Numerical Rating Scale (NRS): mild (NRS 1–3), moderate (NRS 4–6), and severe (NRS 7–10). A statistically significant difference was observed between the mild and severe pain groups (*p* < 0.05, post hoc analysis), with the mild pain group exhibiting higher “maxSCR_CDA” values. No significant differences were detected involving the moderate pain group (ns = not statistically significant). Statistical significance was assessed using one-way ANOVA followed by post hoc pairwise comparisons (Games-Howell test). Only for visualization purposes, extreme outliers were removed from the graph. All HRV parameters (e.g., RMSSD, SDNN, LF, HF, and LF/HF ratio) showed no significant association with pain intensity (*p*-value > 0.1 for all HRV features).

### 3.2. Association with Pain Type

The analysis revealed significant differences across the three baseline pain type categories (nociceptive, neuropathic, and mixed) for multiple EDA parameters. Key significant findings included the number of SCRs for the CDA method (“nSCR_CDA”) (*p* = 0.028), the number of SCRs for the TTP method (“nSCR_TTP”) (*p* = 0.015), the maximum SCR amplitude for the TTP method (“maxSCR_TTP”) (*p* = 0.040), and the mean SCR amplitude for the TTP method (“meanSCR_TTP”) (*p* = 0.048) (Table 2).

**Figure 3:**
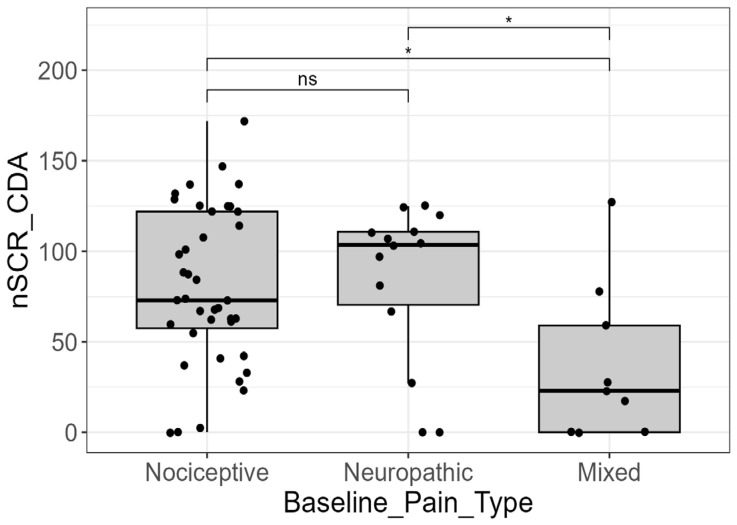
EDA signal and pain types. Distribution of the number of skin conductance responses derived from continuous decomposition analysis (nSR_CDA) across baseline pain types (nociceptive, neuropathic, and mixed). The mixed pain group exhibited a significantly lower number of SCRs compared with both nociceptive and neuropathic pain groups (*p* < 0.05, post hoc pairwise comparisons), indicating an attenuated sympathetic autonomic response. No significant differences were observed between the nociceptive and neuropathic pain groups. Statistical significance was assessed using Kruskal–Wallis test followed by post hoc pairwise comparisons (Dunn’s test). Only for visualization purposes, extreme outliers were removed from the graph. (ns = not significant; * = *p*-value < 0.05).

Specifically, the number of SCRs derived from CDA was found to be a significant parameter in distinguishing between baseline pain types. The pairwise comparisons showed significant differences between the mixed pain group and the other two categories. The mixed pain group exhibited a significantly lower number of SCRs compared to the neuropathic group (*p* = 0.026; adjusted-*p* = 0.038) and the nociceptive group (*p* = 0.009; adjusted-*p* = 0.026). In contrast, no significant difference was observed between the nociceptive and neuropathic groups (*p* = 0.970; adjusted-*p* = 0.970) (Figure 3).

**Figure 4:**
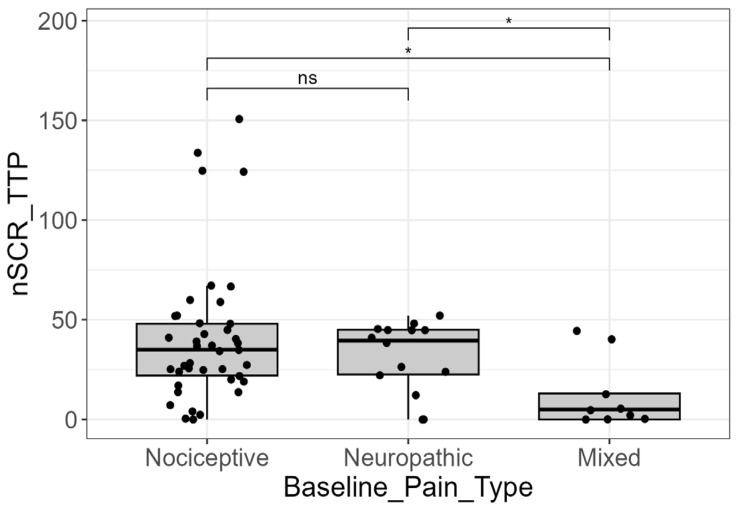
EDA signal and pain types. Distribution of the number of skin conductance responses derived from trough-to-peak analysis (nSCR_TTP) across baseline pain types (nociceptive, neuropathic, and mixed). The mixed pain group exhibited a significantly lower number of SCRs compared with both nociceptive and neuropathic pain groups (*p* < 0.05, post hoc pairwise comparisons), indicating an attenuated sympathetic autonomic response. No statistically significant differences were observed between the nociceptive and neuropathic pain groups. Statistical significance was assessed using Kruskal–Wallis test followed by post-hoc pairwise comparisons (Dunn’s test). Only for visualization purposes, extreme outliers were removed from the graph. (ns = not significant; * = *p*-value < 0.05).

Moreover, the number of SCRs (from TTP analysis ) was significantly lower in the mixed pain group (median = 5.0; IQR = 13.0) when compared to the nociceptive group (median = 35.0; IQR = 26.0; *p* = 0.004; and adjusted-*p* = 0.012) and the neuropathic group (median = 39.5; IQR = 22.5; *p* = 0.024). Consistent with the CDA results, the comparison between the nociceptive and neuropathic groups did not yield a statistically significant difference (*p* = 0.761; adjusted-*p* = 0.761) (Figure 4).

**Figure 5:**
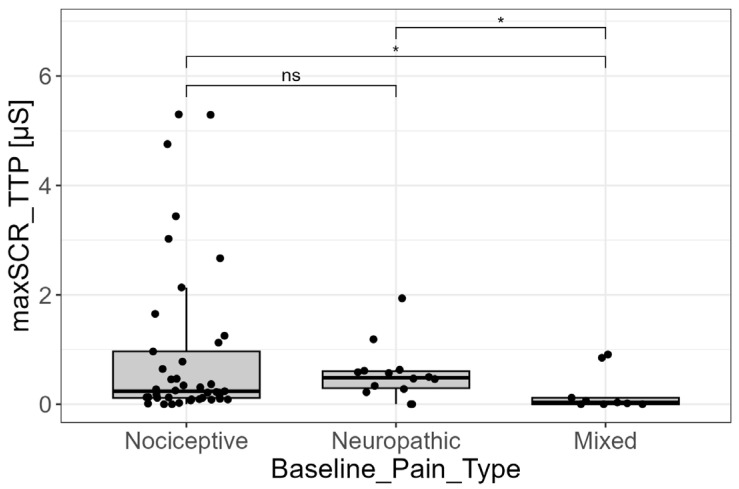
EDA signal and pain types. Analysis of the maximum amplitude of skin conductance responses derived from trough-to-peak analysis (maxSCR_TTP) across the three baseline pain type categories (nociceptive, neuropathic, and mixed). The maxSCR_TTP in the mixed pain group was significantly lower compared with both nociceptive and neuropathic pain groups (*p* < 0.05, post hoc pairwise comparisons). Statistical significance was assessed using Kruskal–Wallis test followed by post hoc pairwise comparisons (Dunn’s test). Only for visualization purposes, extreme outliers were removed from the graph. ns = not statistically significant. (ns = not significant; * = *p*-value < 0.05).

The discrimination by pain type was further supported by the analysis of the maximum SCR amplitude (from TTP analysis). The pairwise comparisons revealed significant differences driven entirely by the mixed pain group. Specifically, the maximum SCR amplitude in the mixed pain group was significantly lower when compared to the nociceptive group (*p* = 0.024; adjusted-*p* = 0.035) and the neuropathic group (*p* = 0.016; adjusted-*p* = 0.035). Conversely, the maximum SCR amplitude did not statistically differ between the nociceptive and neuropathic groups themselves (*p* = 0.531; adjusted-*p* = 0.531) (Figure 5).

**Figure 6:**
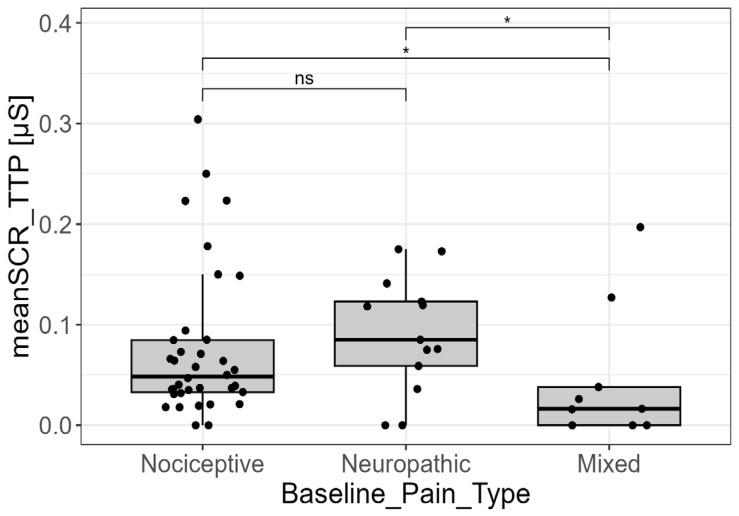
EDA signal and pain types. Analysis of the mean of skin conductance responses amplitude derived from trough-to-peak analysis (meanSCR_TTP) across the three baseline pain type categories (nociceptive, neuropathic, and mixed). The mixed pain group exhibited a lower mean amplitude compared with both nociceptive and neuropathic pain groups (*p* <0.05, post hoc pairwise comparisons). Statistical significance was assessed using Kruskal–Wallis test followed by post hoc pairwise comparisons (Dunn’s test). Only for visualization purposes, extreme outliers were removed from the graph. ns = not statistically significant. (ns = not significant; * = *p*-value < 0.05).

The mean SCR amplitude obtained with the TTP method also proved to be a significant discriminator across the pain type categories. Pairwise comparisons confirmed that the significance was attributed to the mixed pain group exhibiting a lower mean amplitude compared to both the nociceptive group (*p* = 0.030; adjusted-*p* = 0.045) and the neuropathic group (*p* = 0.018; adjusted-*p* = 0.045). The mean SCR amplitude did not significantly differ between the nociceptive and neuropathic groups themselves (*p* = 0.497; adjusted-*p* = 0.497). Furthermore, descriptive analysis showed that the highest mean amplitude value overall was assumed by the nociceptive pain group (Figure 6).

The HRV analysis did not reveal any statistically significant associations with either pain intensity or pain type. Parameters such as SDNN, RMSSD, mean heart rate, and all spectral components exhibited *p*-values well above the significance threshold, indicating that HRV did not discriminate among the clinical pain categories considered in this study (Table 3).

### 3.3. Association Between Biosignals and Other Clinical Variables Across Pain Intensity and Type

Finally, the presence or absence of further specific clinical variables (bone metastases, BCTP, and MED more or less than 60 mg/day) was assessed by conducting a separate statistical analysis on a subset of biosignal features that emerged as the most informative, based on the results shown in the previous sections.

In particular, since the maximum SCR amplitude, as calculated by the CDA method, proved to be a potential distinguishing parameter across pain intensity classes, a subgroup analysis was performed and is shown in Table 4.

No statistically significant trend emerged from the subgroup analysis according to pain intensity classes, as shown in Table 4, nor by grouping cases by BTPC, bone metastases, or MED. Thus, no associations were observed between the above-mentioned clinical variables and the selected biosignal feature.

Conversely, the number of SCR as well as maximum and mean SCR values calculated through the TTP method showed statistically significant association with pain intensity classes, also when grouped according to clinical features (bone metastases, BTPC, and MED) (Table 5).

As from Table 5, mixed pain is well distinguished from nociceptive or neuropathic pain for patients without bone metastases for all the considered subsets of biosignal features. However, the statistical significance is not confirmed for patients with bone metastases. As far as the presence of BTCP, no significant features emerged except that the number of SCR calculated with the CDA method proved to be able to discriminate between nociceptive and mixed pain type for those patients without BTCP. Finally, as far as the MED, nociceptive and mixed appear to be distinguished either by the number of SCR or the maximum SCR amplitude, only for those patients not using higher doses of opioids (i.e., MED > 60 mg/day).

## 4. Discussion

The main objective of this study was to investigate the efficacy of EDA and HRV-derived parameters as objective indicators for discriminating intensity and type of cancer pain. For EDA, the analysis revealed a significant association between maximum SCR amplitude (i.e., MaxCDA), obtained via CDA, and pain intensity categories. Specifically, post hoc analysis indicated a significant difference between patients reporting low-intensity pain and those with high-intensity pain.

Importantly, the present findings should be interpreted in light of the exploratory nature of the study and the specific characteristics of the oncological population examined. The observed association between EDA parameters and pain intensity or pain type does not imply a direct or linear correspondence between subjective pain perception and autonomic activation. Rather, our results suggest that EDA captures pain-related alterations in autonomic regulation that may be modulated by multiple interacting factors, including chronicity of pain, central sensitization, and ongoing pharmacological treatments. In particular, the counterintuitive finding of higher SCR amplitudes in patients reporting lower pain intensity underscores the complexity of interpreting biosignals in cancer pain. This pattern is biologically plausible in the context of chronic pain and advanced disease, where sustained nociceptive input and analgesic exposure may lead to autonomic blunting or reduced sympathetic reactivity. EDA is sensitive to alterations in sympathetic regulation but is strongly influenced by contextual factors and the individual’s physiological state [22]. Therefore, EDA should not be viewed as a direct surrogate of pain intensity, but rather as an indicator of altered autonomic responsiveness associated with different pain phenotypes.

Furthermore, the lack of significant associations between HRV parameters and pain characteristics should not be interpreted as evidence of the irrelevance of cardiac autonomic markers, but probably as a consequence of methodological constraints, including short recording duration and the high interindividual variability typical of cancer patients. Taken together, these findings support a cautious and integrative interpretation, in which biosignals are considered complementary components of a broader, multimodal pain assessment framework rather than standalone diagnostic markers.

Unexpectedly, patients with low baseline pain exhibited higher Max SCR amplitudes compared to those with medium or high pain levels. This counterintuitive finding contrasts with the logical understanding that higher pain intensity correlates with increased sympathetic nervous system arousal and, consequently, larger EDA responses. A plausible explanation is the confounding effect of pharmacological therapy. Patients with greater baseline pain severity are typically treated with more intense pharmacological regimens, including higher doses of opioids and adjuvant analgesics. These medications, particularly opioids, are known to depress the central nervous system and attenuate the physiological responses of the ANS, thus masking the true pain-evoked sympathetic arousal and leading to lower SCR amplitudes in the high-pain group. Moon et al. [23], for example, reported that substance use, including opioids, is associated with significant reductions in HRV and autonomic alterations, indicating that analgesic medications and chronic use can confound autonomic indicators. Nevertheless, investigating correlations between biosignals and pain in oncology patients remains inherently challenging. Previously, in a multimodal analysis, we failed to find a significant correlation between subjective and objective pain variables [18].

Beyond intensity, EDA parameters demonstrated significant capacity to discriminate between different pain types. The number of SCRs (from both CDA and TTP analyses) and the maximum/mean SCR amplitudes (from TTP analysis) were significantly associated with pain classification (*p* < 0.05). In particular, patients with mixed pain (a combination of nociceptive and neuropathic components) showed a significantly lower number of SCRs and lower SCR amplitudes compared to patients with purely nociceptive or purely neuropathic pain. This suggests that the physiological signature of mixed pain, possibly due to complex central and peripheral interactions, may result in a dampened or disorganized sympathetic output as captured by the EDA response [24]. Furthermore, the study confirmed that the presence of BTCP did not significantly alter the measured EDA or HRV parameters. It may suggest that physiological markers reflect the underlying baseline or chronic pain state rather than transient exacerbations [18]. Moreover, since BTCP was assessed at baseline, the lack of association between this pain phenomenon and biosignals may reflect the fact that physiological recordings captured baseline autonomic activity rather than transient pain flares.

### Strengths and Limitations

A major strength of the present study lies in the investigation of ANS responses to cancer-related pain in a real-world clinical setting, rather than in experimentally induced pain paradigms. Notably, by integrating EDA and ECG-derived parameters with clinical pain characterization, this work provides clinically grounded evidence on the feasibility and limits of biosignal-based pain assessment in oncology. Importantly, the analysis focused not only on pain intensity but also on pain type, offering novel insights into the potential role of EDA in distinguishing pain phenotypes (especially mixed pain) in cancer patients.

Several study limitations affect the results. A major limitation is the potential confounding effect of pharmacological treatments on the ANS activity. This study did not fully account for all concomitant medications known to impact ANS responses. Specifically, data regarding the use and dosage of drugs such as beta-blockers, tricyclic antidepressants, selective serotonin reuptake inhibitors (SSRIs), and other medications were not integrated into the analysis. These drugs can significantly attenuate or modulate sympathetic tone, potentially masking the true physiological arousal evoked by pain. For example, SSRIs have been associated with reduced SCRs, and tricyclic antidepressants with altered autonomic cardiac indices, indicating that psychotropic medications can directly influence measures of autonomic activity such as EDA and HRV [25]. Consequently, in our analysis, this confounding effect likely contributed to the unexpected finding that cancer patients suffering from severe pain exhibited lower EDA amplitudes (MaxCDA) than those in the mild pain group. Additionally, other limitations concern the adopted methodology. In contrast to EDA, the analysis revealed no statistically significant associations between any extracted HRV feature (in both time and frequency domains, including SDNN, RMSSD, and LF/HF ratio) and either pain intensity or pain type. While previous studies indicated HRV as a useful marker for nociceptive responses in experimentally induced acute pain in healthy adults [13], in our investigation, its lack of utility is likely a consequence of the short recording duration. HRV requires longer recording periods, ideally at least ten minutes, for robust frequency domain analysis. Nevertheless, this approach was not feasible in all oncology patients due to the study constraints. In our study, all acquisitions were truncated to the shortest recording length of three minutes to ensure uniform processing. This short duration significantly limited the reliability of the calculated HRV features, particularly those in the very LF domain. Additionally, due to the limited sample size and heterogeneity of cancer diagnoses, stratified analyses according to tumor type were not feasible. Recent large-scale evidence indicates that HRV reduction in cancer patients tends to occur uniformly across different tumor locations and progressively worsens with disease stage, rather than reflecting cancer-specific or site-specific autonomic signatures. In a cohort of nearly 800 patients, Ben-David et al. [26] demonstrated that HRV metrics were consistently reduced across breast, gastrointestinal, genitourinary, and respiratory cancers, with no significant differences attributable to tumor location, while disease progression emerged as the dominant determinant of autonomic impairment. These findings suggest that HRV primarily reflects a global cancer-related autonomic dysregulation rather than localized or phenotype-specific processes.

Finally, although pain duration, opioid therapy, and oncological treatments may significantly influence autonomic responses [23,27], the present study was not powered to perform stratified analyses based on these variables.

According to Moscato et al. [7], to address future developments in this field, research should prioritize using wearable technology for continuous monitoring in natural environments. Moreover, it is necessary to adopt large-scale data methodologies, potentially enhanced by artificial intelligence (AI) [28], to incorporate various stratification variables, such as the specific type and stage of cancer, anticancer therapy including immune checkpoint inhibitors [29], and relevant demographic or psychosocial factors, alongside more standardized recording protocols. This approach will be fundamental to better explain the complexity of cancer pain and to achieve a more precise characterization of nociceptive and neuropathic pain phenotypes, as well as features of other crucial cancer pain manifestations such as BTCP [30].

## 5. Conclusions

Given the findings, EDA and its distinct parameters (Max SCR Amplitude, SCR count) may represent promising objective biomarkers for cancer pain assessment, particularly for discriminating among pain types. The observed inverse relationship between high pain intensity and Max SCR Amplitude warrants further investigation with rigorous control, including analgesic medication dosage and other covariates. The lack of significant results for HRV in this context primarily highlights the methodological limitation of short recording windows in chronic pain populations. Given the potential of AI strategies, future work should focus on developing multimodal frameworks that optimally integrate EDA and HRV with other robust physiological signals, such as EEG, to improve the sensitivity and specificity of APA and, ultimately, to provide useful support tools for the clinical management of oncological patients.

## Figures and Tables

**Table 1:** Demographic data and variables (*n* = 64).

Variable		
Age (years)	Mean (SD)	61 (13.3)
Gender, *n* (%)	Male	41 (64)
	Female	23 (36)
BMI (kg/m^2^)	Mean (SD)	25 (4.54)
Cancer Type, *n* (%)	Breast	6 (9.4)
	Gastrointestinal	11 (17.2)
	Lung	10 (15.6)
	Bone/Soft Tissue	11 (17.2)
	Others	26 (40.6)
ECOG PS, *n* (%)	ECOG 1	33 (51.6)
	ECOG 2	11 (17.2)
	ECOG 3	7 (10.9)
	ECOG 4	13 (20.3)
Baseline Pain Intensity (NRS), *n* (%)	Mild (1–3)	19 (30.2)
	Medium (4–6)	16 (25.4)
	High (7–10)	28 (44.4)
Baseline Pain Type, *n* (%)	Nociceptive	41 (64.1)
	Neuropathic	14 (21.9)
	Mixed	9 (14.1)
BTCP, *n* (%)	Yes	28 (43.8)
Bone Metastases, *n* (%)	Yes	23 (35.9)
MED, *n* (%)	Yes	34 (53.1)

Abbreviations: ECOG PS, Eastern Cooperative Oncology Group Performance Status; NRS, numeric rating scale; BTCP, breakthrough cancer pain; MED, morphine equivalent dose.

**Table 2:** ANOVA results for EDA parameters by baseline pain type.

EDA Parameter	Statistic	*p*-Value
Number of SCR with the CDA method	7.17	0.028
Number of SCR with the TTP method	8.33	0.015
Max SCR Amplitude with TTP method	6.42	0.040
Mean SCR Amplitude with TTP method	6.06	0.048

**Table 3:** Statistical analysis (Kruskal–Wallis test) on HRV.

HRV Parameter	Domain	*p*-Value vs. Pain Intensity	*p*-Value vs. Pain Type
RMSSD	Time	0.374	0.333
SDNN	Time	0.172	0.224
Mean HR	Time	0.642	0.897
VLF Power	Frequency	0.395	0.280
LF Power	Frequency	0.325	0.377
HF Power	Frequency	0.987	0.804
LF/HF Ratio (Sympatho-Vagal Balance)	Frequency	0.272	0.266

Abbreviations: RMSSD, Root Mean Square of Successive Differences; SDNN, Standard Deviation of NN intervals; HR, heart rate; VLF, very low frequency; LF, low frequency; HF, high frequency.

**Table 4:** Statistical analysis (Kruskal–Wallis test and Dunn’s post hoc test) on selected EDA features across pain intensity categories and clinical grouping factors.

EDA Parameter	Group	*p*-Value vs. Pain Intensity	Statistically Significant Subgroups
Max SCR Amplitude with CDA method	Bone metastases = yes	0.111	-
	Bone metastases = no	0.221	-
	BTCP = yes	0.129	-
	BTCP = no	0.068	-
	MED > 60 mg/day	0.283	-
	MED < 60 mg/day	0.084	-

Abbreviations: EDA, electrodermal activity; SCR, skin conductance response; CDA, continuous decomposition analysis; BTCP, Breakthrough cancer pain; MED, morphine equivalent dose

**Table 5:** Statistical analysis (Kruskal–Wallis test and Dunn’s post hoc test) on selected EDA features across pain type categories and clinical grouping factors (bold font is used to highlight statistically significant results, *p*-value<0.05; adj.*p* = adjusted *p*-value).

EDA Parameter	Group	*p*-Value vs. Pain Type	Statistically Significant Subgroups
Number of SCR with the CDA method	Bone metastases = yes	0.664	-
	Bone metastases = no	**0.014**	Nociceptive-Mixed (adj.*p* = 0.010) Neuropathic-Mixed (adj.*p* = 0.024)
Number of SCR with the TTP method	Bone metastases = yes	0.475	-
	Bone metastases = no	**0.015**	Nociceptive-Mixed (adj.*p* = 0.013) Neuropathic-Mixed (adj.*p* = 0.016)
Max SCR Amplitude with TTP method	Bone metastases = yes	0.473	-
	Bone metastases = no	**0.016**	Nociceptive-Mixed (adj.*p* = 0.014) Neuropathic-Mixed (adj.*p* = 0.016)
Mean SCR Amplitude with TTP method	Bone metastases = yes	0.175	-
	Bone metastases = no	**0.035**	Nociceptive-Mixed (adj.*p* = 0.030) Neuropathic-Mixed (adj.*p* = 0.045)
Number of SCR with the CDA method	BTCP = yes	0.138	-
	BTCP = no	**0.037**	Nociceptive-Mixed (adj.*p* = 0.037)
Number of SCR with the TTP method	BTCP = yes	0.088	-
	BTCP = no	0.064	-
Max SCR Amplitude with TTP method	BTCP = yes	0.139	-
	BTCP = no	0.238	-
Mean SCR Amplitude with TTP method	BTCP = yes	0.058	-
	BTCP = no	0.246	-
Number of SCR with the CDA method	MED > 60 mg/day	0.251	-
	MED < 60 mg/day	**0.038**	Nociceptive-Mixed (adj.*p* = 0.031)
Number of SCR with the TTP method	MED > 60 mg/day	0.151	-
	MED < 60 mg/day	**0.046**	Nociceptive-Mixed (adj.*p* = 0.039)
Max SCR Amplitude with TTP method	MED > 60 mg/day	0.328	-
	MED < 60 mg/day	**0.041**	Nociceptive-Mixed (adj.*p* = 0.038) Neuropathic-Mixed (adj.*p* = 0.038)
Mean SCR Amplitude with TTP method	MED > 60 mg/day	0.156	-
	MED < 60 mg/day	0.092	-

Abbreviations: SCR, skin conductance response; CDA, continuous decomposition analysis; TTP, trough-to-peak; BTCP, Breakthrough cancer pain; MED, morphine equivalent dose

## Data Availability

All data are available here and at the link https://doi.org/10.5281/zenodo.13711426.
